# Mammary adipocytes protect triple-negative breast cancer cells from ferroptosis

**DOI:** 10.1186/s13045-022-01297-1

**Published:** 2022-06-03

**Authors:** Yizhao Xie, Biyun Wang, Yannan Zhao, Zhonghua Tao, Ye Wang, Guangliang Chen, Xichun Hu

**Affiliations:** 1 Department of Breast Cancer and Urological Medical Oncology, Fudan University Shanghai Cancer Center, 270, Dong’an Road, Xuhui District, Shanghai, 200032 China; 2 Department of Oncology, Shanghai Medical College, Fudan University, Shanghai, China; 3 Department of Lymphoma, Fudan University Shanghai Cancer Center, 270, Dong’an Road, Xuhui District, Shanghai, 200032 China

**Keywords:** Breast cancer, Adipocytes, Ferroptosis, Cell death, Lipid metabolism

## Abstract

Ferroptosis, a novel non-apoptotic form of cell death, can induce tumor cell death and treatment resistance. Lipid metabolism is closely related to ferroptosis; however, the effect of mammary adipocytes on breast cancer ferroptosis remains to be elucidated. Here, we established the co-culture system of adipocyte-breast cancer cells and revealed the protection of triple-negative breast cancer from ferroptosis by adipocytes. Then, we performed the lipidomics analysis comparing lipid metabolites of co-cultured and normal-cultured cells. Mechanistically, oleic acid secreted from adipocytes inhibited lipid peroxidation and ferroptosis of triple-negative breast cancer cells in the presence of ACSL3. Taken together, mammary adipocytes can protect breast cancer cells from ferroptosis through oleic acid in the presence of ACSL3. These findings could provide new ideas and targets for tumor treatment.

**Supplementary Information:**

The online version contains supplementary material available at 10.1186/s13045-022-01297-1.

## To the editor

Breast cancer cells survive in mammary adipocytes, which provides a unique micro-environment for neoplasia cells. A remodeling effect of adipocytes on breast cancer lipid metabolism was observed and free fatty acids (FFA) and lipid droplets could make breast cancer cells more invasive [1, 2]. Ferroptosis is a non-apoptotic form of cell death driven by excessive lipid peroxidation, which was involved in the pathogenesis, development as well as therapeutic targets of multiple types of cancer including triple-negative breast cancer (TNBC) [3, 4]. Studies found that polyunsaturated fatty acids (PUFAs) could induce peroxidation of lipid bilayer while exogeneous monounsaturated fatty acids (MUFAs) could inhibit ferroptosis [5, 6]. However, the effect of mammary adipocytes on TNBC ferroptosis remains unknown.

**Fig. 1:**
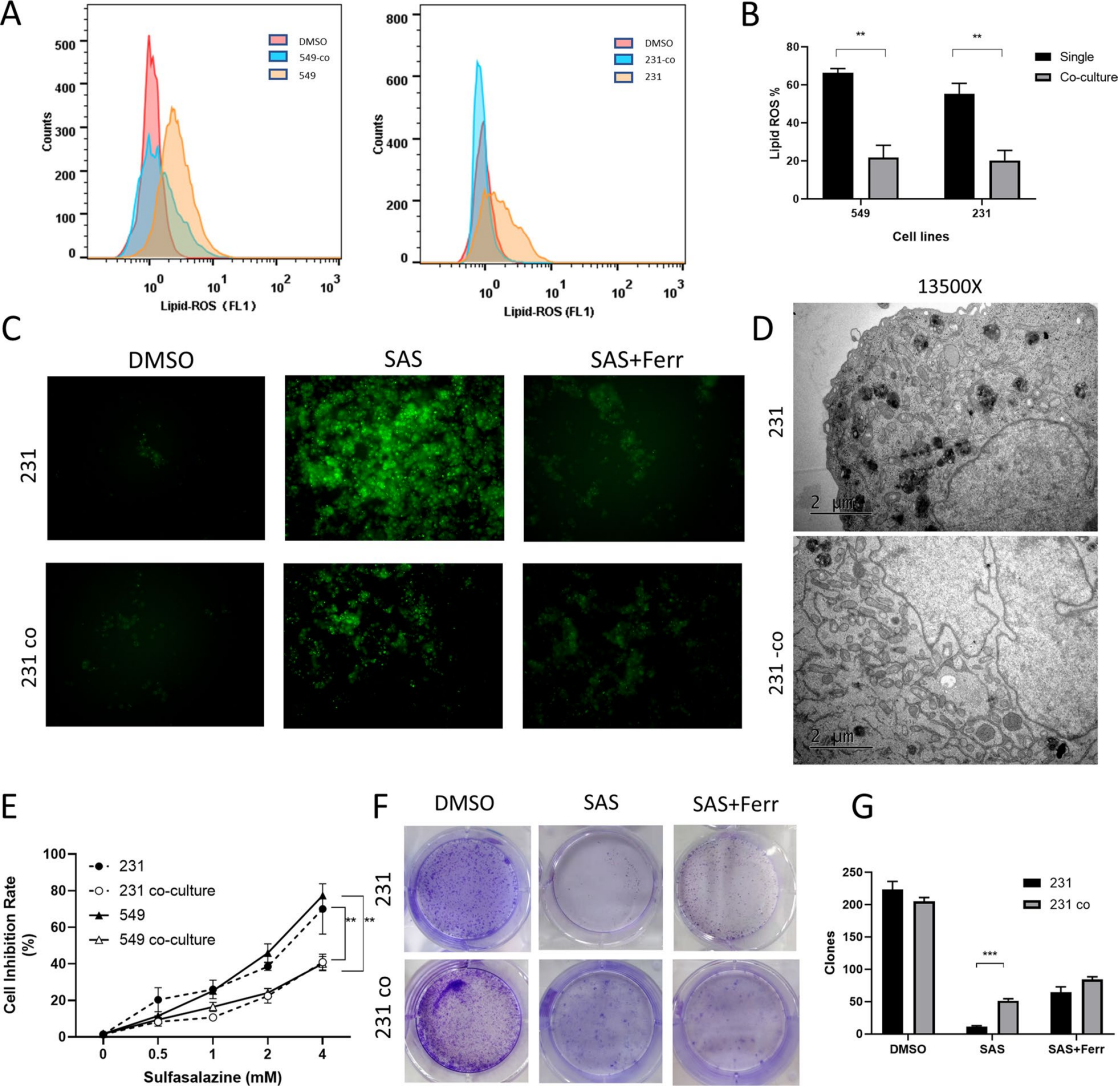
Mammary adipocytes protect breast cancer cells from ferroptosis. **A** Lipid peroxidation levels in co-culture and control group of MDA-MB-231 and BT-549 cells treated with DMSO or SAS (2 mM). **B** Quantitative analysis of lipid peroxidation levels of co-culture and control cells treated with DMSO or SAS (2 mM). **C** Fluorescence microscopy images of co-culture and control cells treated with DMSO or SAS or SAS in combination with ferrostatin-1 (5 μM). **D** TEM images of co-culture and control cells treated with SAS. **E** Cell viability in co-culture and control group of MDA-MB-231 and BT-549 cells treated with indicated concentrations of SAS. **F** Representative images of clonogenic assay in co-culture or control group of cells subjected to the indicated treatments. **G** Quantification of clonogenic survival fractions in cells subjected to the indicated treatments

We first established the mammary adipocyte-breast cancer cell co-culture system. Mature adipocytes were derived and separated from patients underwent breast cancer surgery and an isolated co-culture system was established using transwell culture dish where adipocyte was in upper level and breast cancer cell was in lower level (Additional file 1: Fig. S1). After 72 h co-culture, red oil staining and Nile red staining were performed showing more lipid droplets in co-culture group than in regular culture group (Additional file 1: Fig. S1). Then, we choose sulfasalazine as ferroptosis inducer and BODIPY™ 581/591C11 staining as detection of level of lipid peroxidation according to previous data [7–9]. We found that both in TNBC cell lines BT-549 and MDA-MB-231, increased lipid-ROS levels derived from ferroptosis could be induced by Sulfasalazine (SAS), and co-cultured cells significantly inhibited lipid peroxidation detected by flow cytometry compared to normal-cultured cells and was partly rescued by concurrent administration of ferroptosis inhibitor, ferrostatin-1 (Fig. 1A–C). Transmission electron microscope (TEM) assay also found a marked increased mitochondrial peroxidation of normal-cultured cells compared to co-cultured cells after treatment of SAS (Fig. 1D, Additional file 2: Fig. S2). Moreover, results showed a significantly lower inhibition rate as well as a significantly higher clone numbers of co-cultured cells compared to normal-cultured cells after treatment of SAS and the growth inhibition could also be partially restored by ferrostatin-1 (Fig. 1E–G). We also introduced apoptosis inhibitor Z-VAD-FMK and autophagy inhibitor 3-MA and found they cannot restore the cell viability of SAS treated cells compared to ferrostatin-1 (Additional file 2: Fig. S2). We used another type of ferroptosis inducer RSL3 to confirm our results (Additional file 2: Fig. S2). These findings suggest an inhibitory effect of adipocytes on ferroptosis of breast cancer cells.

**Fig. 2:**
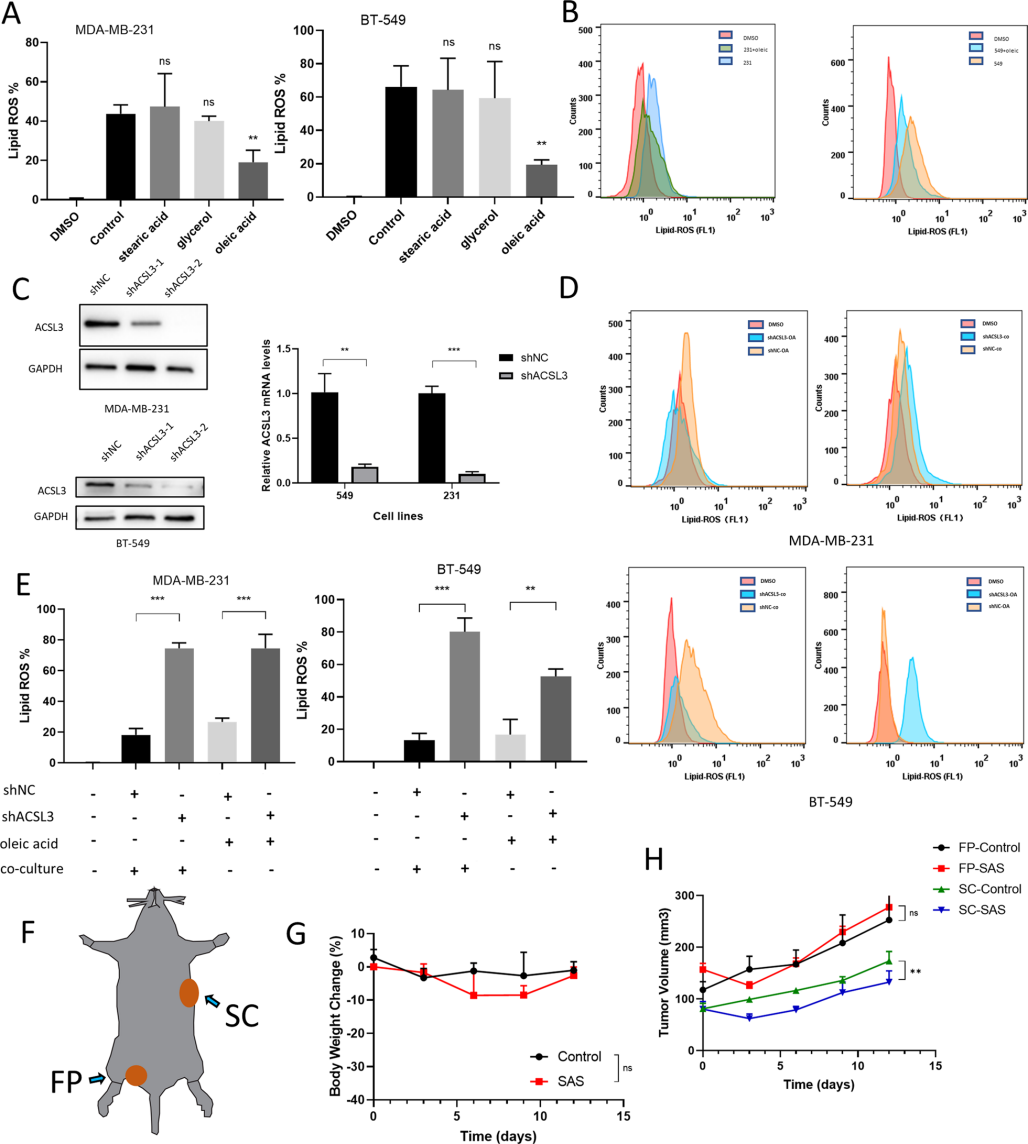
Adipocytes protect breast cancer cells from ferroptosis through oleic acid in the presence of ACSL3. **A** Lipid peroxidation levels in MDA-MB-231 and BT-549 cells co-cultured with exogenous fatty metabolites oleic acid (OA, 500 μM) and treated with SAS or DMSO. **B** Quantitative analysis of lipid peroxidation levels in cells co-cultured with exogenous fatty metabolites OA (500 μM), stearic acid (25 μM), glycerol (500 μM) and DMSO, then treated with SAS or DMSO. **C** The mRNA and protein expression levels of ACSL3 confirmed by qRT-PCR and western blotting in MDA-MB-231 and BT-549 cells expressing control vector (shNC) or sh-ACSL3. **D** Lipid peroxidation levels in shACSL3 and shNC MDA-MB-231 and BT-549 cells co-cultured with adipocytes or OA and treated with DMSO or SAS. **E** Quantitative analysis of lipid peroxidation levels in shACSL3 and shNC cells co-cultured with adipocytes or OA and treated with DMSO or SAS. **F** Schematic diagram showing nude mice bearing MDA-MB-231 xenografts at fat pad (FP) of left groin and right back subcutaneously (SC). **G** Body weights of nude mice bearing xenografts treated with SAS, P values were calculated by 2-tailed unpaired Student’s t-test. **H** Volume of MDA-MB-231 xenografts at FP or SC treated with normal saline (control) or SAS at different time points. P values determined using 2-way ANOVA

In order to investigate the mechanisms by which adipocytes inhibit ferroptosis, we firstly conducted a lipidomic analysis on co-cultured and normal-cultured MDA-MB-231 cell lines. Univariate analysis indicated 88 upregulated metabolites and 12 downregulated metabolites in co-cultured cells. The detailed results and expression levels were shown in Additional file 3: Fig. S3 and Tables S1, S2. In brief, the lipidomic analysis suggested a great elevation of phospholipid biosynthesis pathway of co-cultured cells compared to normal-cultured cells. These findings further suggest us adipocytes inhibit ferroptosis through metabolites related to phospholipid biosynthesis with regards to the vital role of acid-containing phospholipids in ferroptosis. We next found a significant higher non-esterified fatty acids (NEFA) levels in co-cultured cell lines compared to normal-cultured cell lines (Additional file 4: Fig. S4). After introduction of exogenous glycerol, oleic acid (MUFA) and stearic acid (PUFA), a significant inhibition of lipid-ROS levels in exogenous oleic acid group compared to control group was observed (Fig. 2A, B). Cell viability tests and clonal formation showed similar results (Additional file 4: Fig. S4). Previous study suggested that exogenous MUFA could decrease the sensitivity of plasma membrane lipids to lethal oxidation in an acyl-CoA synthetase long-chain family member 3 (ACSL3)-dependent manner among fibrosarcoma cells as well as melanoma cells [5]. Thus, we performed ACSL3-knockdown cell lines using shRNA and found that the lipid-ROS levels of sh-ACSL3 showed a reverse of protection against ferroptosis in adipocyte co-cultured group and exogenous MUFA group, with regards to BT-549 and MDA-MB-231 cell lines (Fig. 2C–E). Furthermore, cell viability tests and clonal formation showed similar results considering shACSL3 cell lines (Additional file 5: Fig. S5). Last but not least, for in vivo study, we inoculated MDA-MB-231 cells into fat pad (FP) of left groin and right back subcutaneously (SC) of nude mice, as the schematic diagram showed (Fig. 2F). Then, the mice were treated with SAS. Mouse weight was monitored and no significant difference was observed in treatment groups and control group throughout the dosing period, indicating a good tolerance (Fig. 2G). Consistent with our in vitro outcomes, the ferroptosis inducer SAS significantly reduced tumor growth in SC group instead of FP group, indicating a protection of adipocytes against ferroptosis (Fig. 2H). Moreover, we found increased lipids in FP tumor using oil-red staining, increased mitochondrial injury in SC tumor using TEM and decreased glutathione peroxidase 4 (GPX4) and solute carrier family 7 member 11 (SLC7A11) levels in SC tumor, which further demonstrated our points (Additional file 6: Fig. S6). Interestingly, previous study found that lymph nodes could protect melanoma cells from ferroptosis by production of MUFA, thus increasing tumor metastasis, which to some extent was conformed to our findings [10]. As more and more clinical trials have been carried out with regards to ferroptosis inducers among cancer patients, our findings could provide valuable information for the benefit of patients.

In conclusion, the present study reveals the protection of adipocytes against breast cancer ferroptosis through MUFA production requiring ACSL3. We also give map to lipidomics of adipocytes co-cultured cells. These findings could provide new ideas and targets for tumor treatment.

## Supplementary Information

**Additional file 1** Fig. S1. Adipocyte-breast cancer cell co-culture system.** A** Schematic diagram of co-culture system.** B** Oil Red staining of co-culture group and regular culture group.** C** Nile red staining of co-culture group and regular culture group

**Additional file 2** Fig. S2.** A** Cell viability in control or co-cultured MDA-MB-231 cells treated with SAS, SAS+Ferrostatin-1, and in control cells treated with SAS+Z-VAD-FMK or SAS+3-MA.** B** Cell viability in control or co-cultured MDA-MB-231 cells treated with RSL3 or RSL3+ Ferrostatin-1.** C** Supporting TEM images of co-culture and control cells treated with SAS.

**Additional file 3** Fig. S3.** A** Lipidomic analysis on co-cultured and normal-cultured MDA-MB-231 cell lines.** B** Volcano plot of univariate statistics.** C** Venn plot of differential metabolites.** D** Boxplot of top 9 differential metabolites ordered by P value. **E** Bubble plot of pathway analysis using SMPDB database. **F** Bar plot of pathway analysis by the HSA set in KEGG.

**Additional file 4** Fig. S4.** A** NEFA levels in culture supernatants from control and co-culture MDA-MB-231 and BT-549 cells.** B** Cell viability in MDA-MB-231 and BT-549 cells co-cultured with indicated fatty metabolites or DMSO, then treated with SAS.** C** Representative images of clonogenic assay in MDA-MB-231 and BT-549 cells co-cultured with indicated fatty metabolites and treated with DMSO, SAS or SAS + Ferrostattin-1.

**Additional file 5** Fig. S5.** A** Cell viability in shACSL3 and shNC MDA-MB-231 and BT-549 cells co-cultured with adipocytes or OA and then treated with SAS.** B** Representative images of clonogenic assay in shACSL3 and shNC cells co-cultured with adipocytes or OA and treated with DMSO, SAS or SAS + Ferrostattin-1.

**Additional file 6** Fig. S6. Supporting figures of mice tumor tissue.** A** Protein levels of GPX4 and SLC7A11 were analyzed by western blotting in tumor of SC and FP group treated with SAS.** B** Adipose infiltration levels tested by Oil Red staining as well as TEM of resected tumor in FP and SC group treated with SAS.

**Additional file 7** Table S1. Expression levels of different metabolites.

**Additional file 8** Table S2. Pathway enrichments of elevated metabolites.

**Additional file 9** Material and Methods.

## Data Availability

The datasets collected and/or analyzed during this study are not publicly available due to hospital policies but are available from the corresponding author on reasonable request.

## Abbreviations

TNBC: Triple-negative breast cancer
MBC: Metastatic breast cancer
HER2: Human epidermal growth factor receptor 2
ER: Estrogen receptor
PR: Progesterone receptor
FFA: Free fatty acids
GPX4: System XC-L-Glutathione reduced-glutathione peroxidase 4
ACSL3: Acyl-CoA synthetase long-chain family member 3
ACSL4: Acyl-CoA synthetase long-chain family member 4
MUFA: Exogeneous monounsaturated fatty acids
TEM: Transmission electron microscope
SAS: Sulfasalazine
PCA: Principal component analysis
DPLA-DS: Orthogonal partial least-squares discrimination analysis
NEFA: Non-esterified fatty acids
FP: Fat pad
SC: Subcutaneous
PUFA-PL: Polyunsaturated fatty acid-containing phospholipids
GPX4: Glutathione peroxidase 4
SLC7A11: Solute carrier family 7 member 11
