# Supplementary figures and images for: Mammary adipocytes protect triple-negative breast cancer cells from ferroptosis
Source: J Hematol Oncol. 2022 Jun 3;15:72. doi: 10.1186/s13045-022-01297-1 (PMC9164506; doi:10.1186/s13045-022-01297-1)

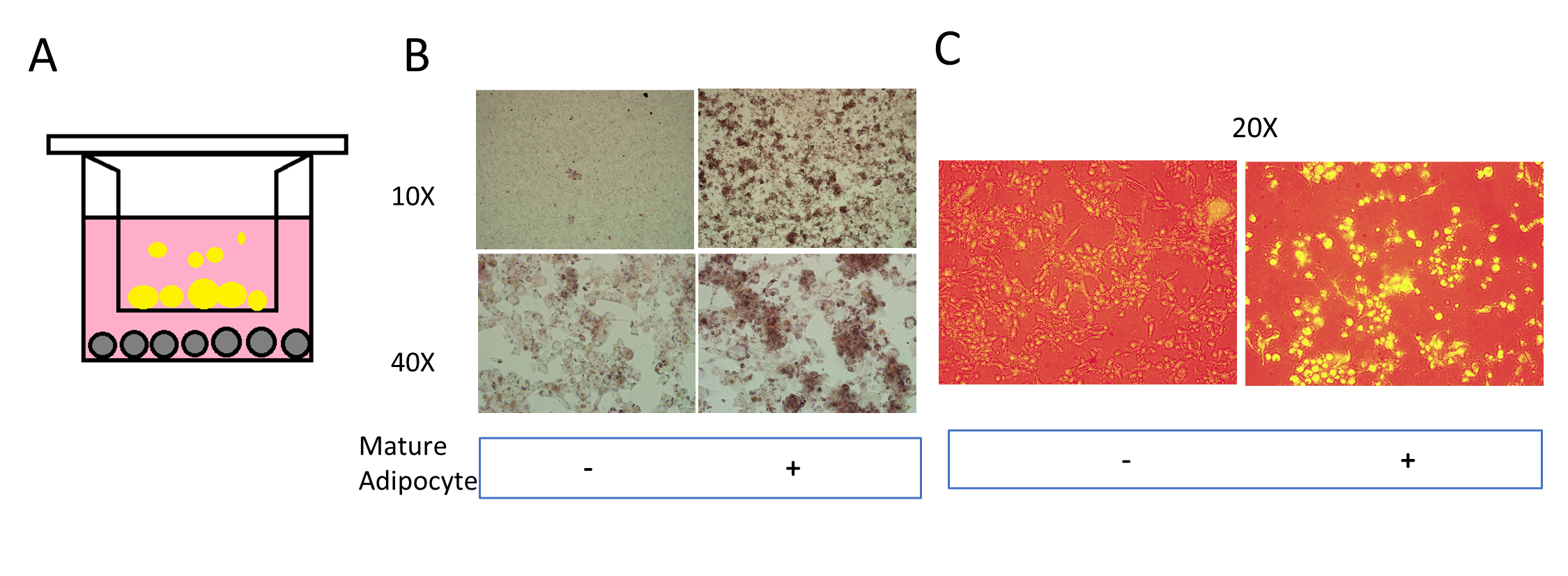

Supplement: Supplementary file 1 — Additional file 1 Fig. S1. Adipocyte-breast cancer cell co-culture system. A Schematic diagram of co-culture system. B Oil Red staining of co-culture group and regular culture group. C Nile red staining of co-culture group and regular culture group [file 13045_2022_1297_MOESM1_ESM.tif]

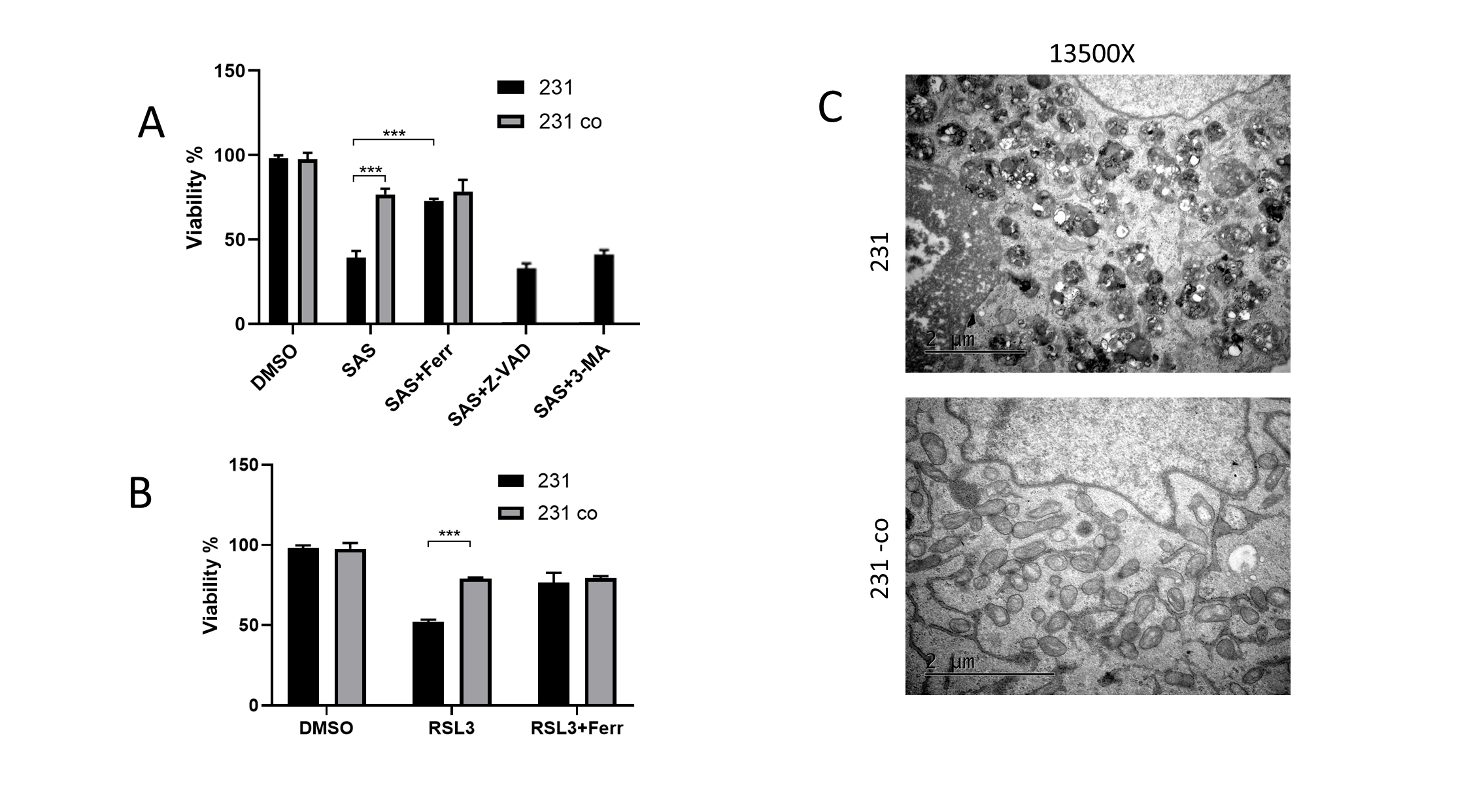

Supplement: Supplementary file 2 — Additional file 2 Fig. S2. A Cell viability in control or co-cultured MDA-MB-231 cells treated with SAS, SAS+Ferrostatin-1, and in control cells treated with SAS+Z-VAD-FMK or SAS+3-MA. B Cell viability in control or co-cultured MDA-MB-231 cells treated with RSL3 or RSL3+ Ferrostatin-1. C Supporting TEM images of co-culture and control cells treated with SAS. [file 13045_2022_1297_MOESM2_ESM.tif]

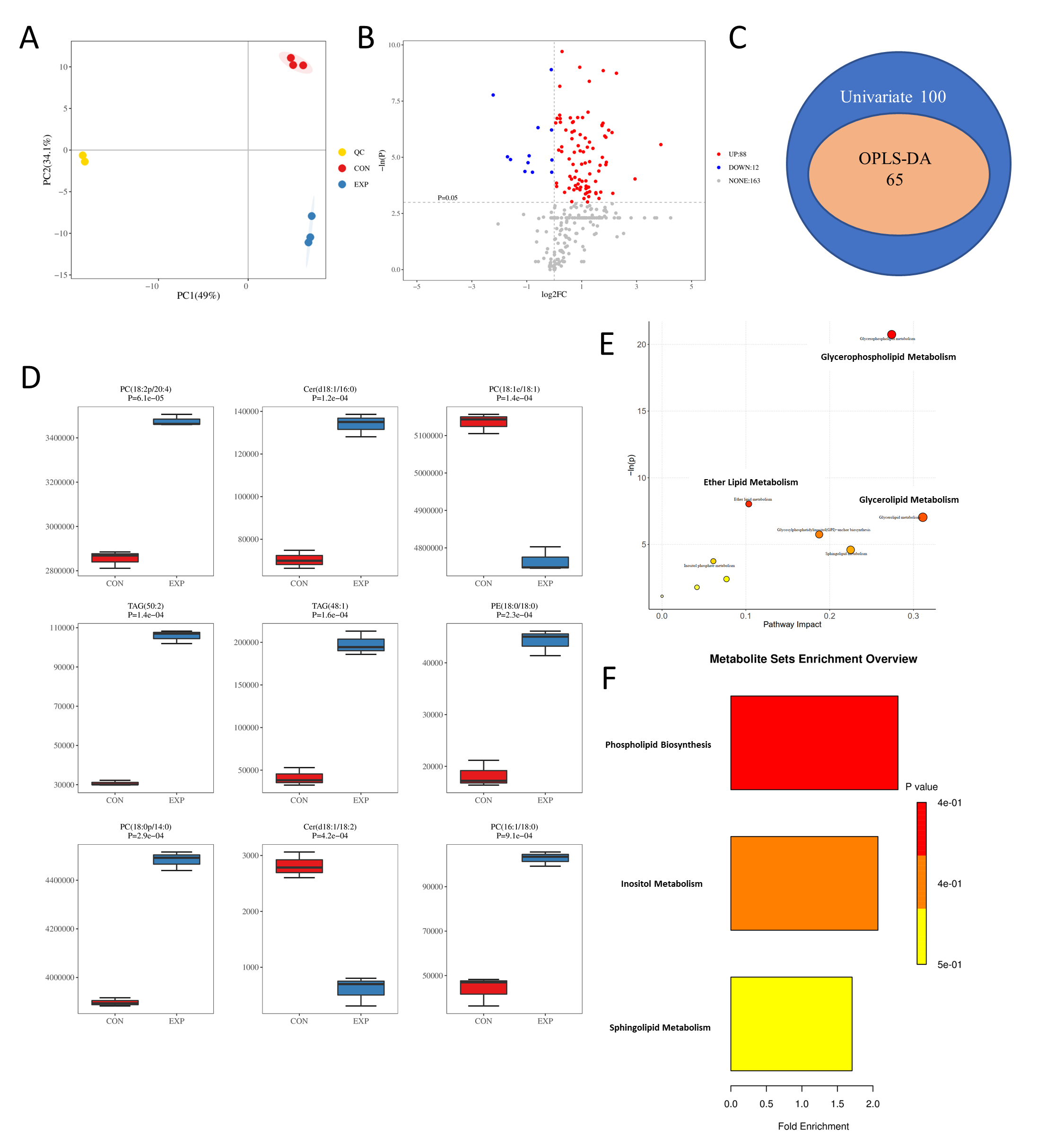

Supplement: Supplementary file 3 — Additional file 3 Fig. S3. A Lipidomic analysis on co-cultured and normal-cultured MDA-MB-231 cell lines. B Volcano plot of univariate statistics. C Venn plot of differential metabolites. D Boxplot of top 9 differential metabolites ordered by P value. E Bubble plot of pathway analysis using SMPDB database. F Bar plot of pathway analysis by the HSA set in KEGG. [file 13045_2022_1297_MOESM3_ESM.tif]

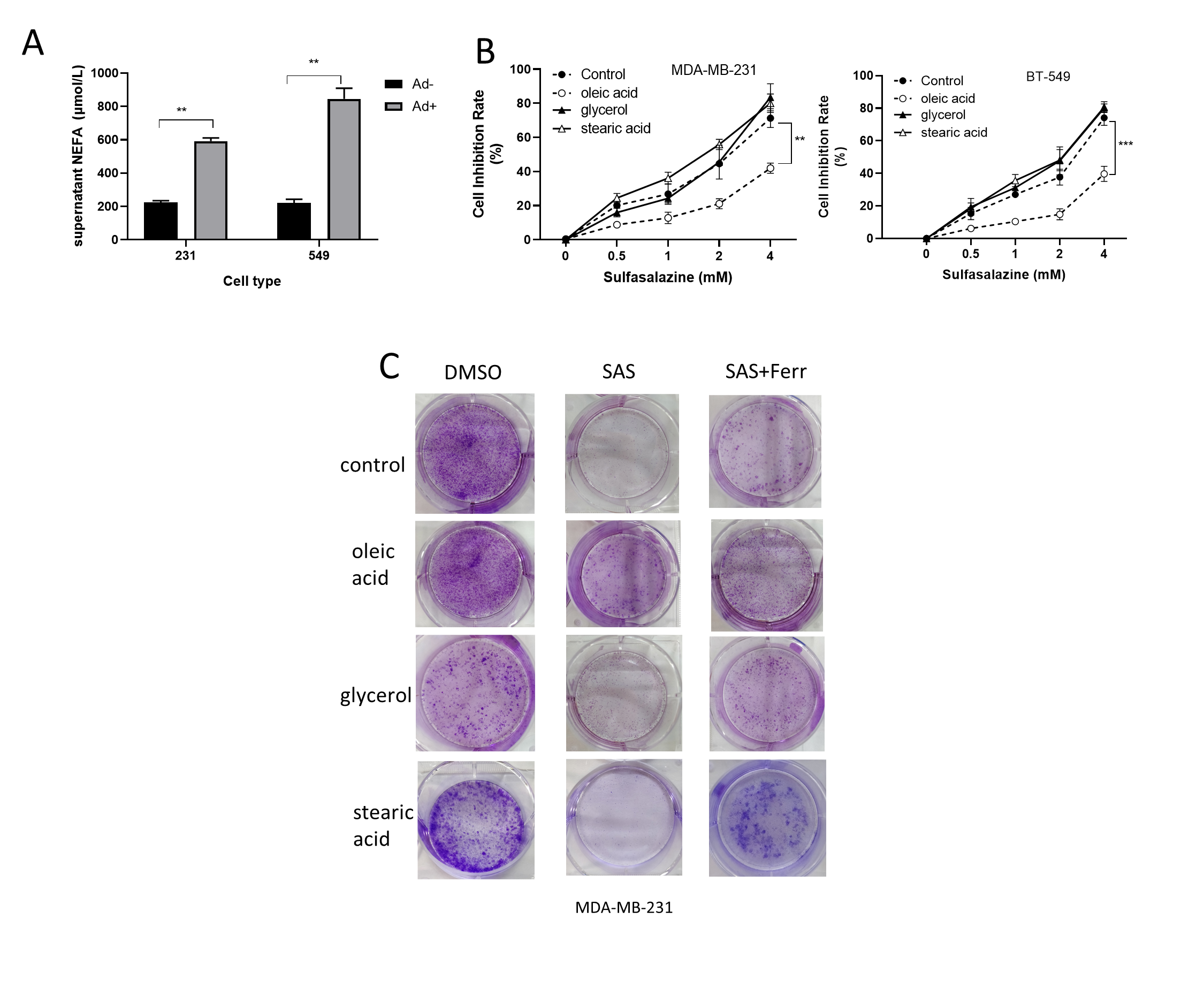

Supplement: Supplementary file 4 — Additional file 4 Fig. S4. A NEFA levels in culture supernatants from control and co-culture MDA-MB-231 and BT-549 cells. B Cell viability in MDA-MB-231 and BT-549 cells co-cultured with indicated fatty metabolites or DMSO, then treated with SAS. C Representative images of clonogenic assay in MDA-MB-231 and BT-549 cells co-cultured with indicated fatty metabolites and treated with DMSO, SAS or SAS + Ferrostattin-1. [file 13045_2022_1297_MOESM4_ESM.tif]

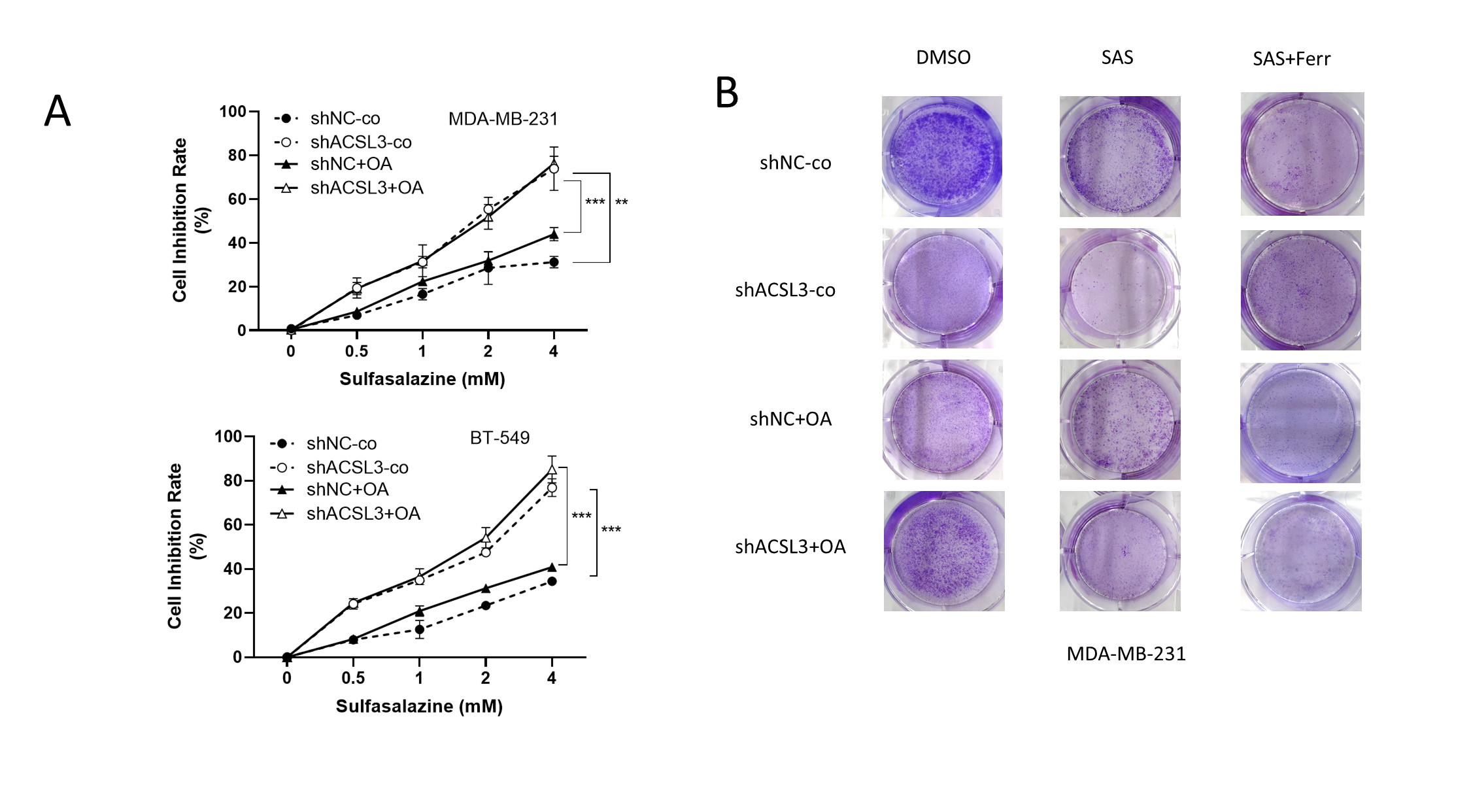

Supplement: Supplementary file 5 — Additional file 5 Fig. S5. A Cell viability in shACSL3 and shNC MDA-MB-231 and BT-549 cells co-cultured with adipocytes or OA and then treated with SAS. B Representative images of clonogenic assay in shACSL3 and shNC cells co-cultured with adipocytes or OA and treated with DMSO, SAS or SAS + Ferrostattin-1. [file 13045_2022_1297_MOESM5_ESM.tif]

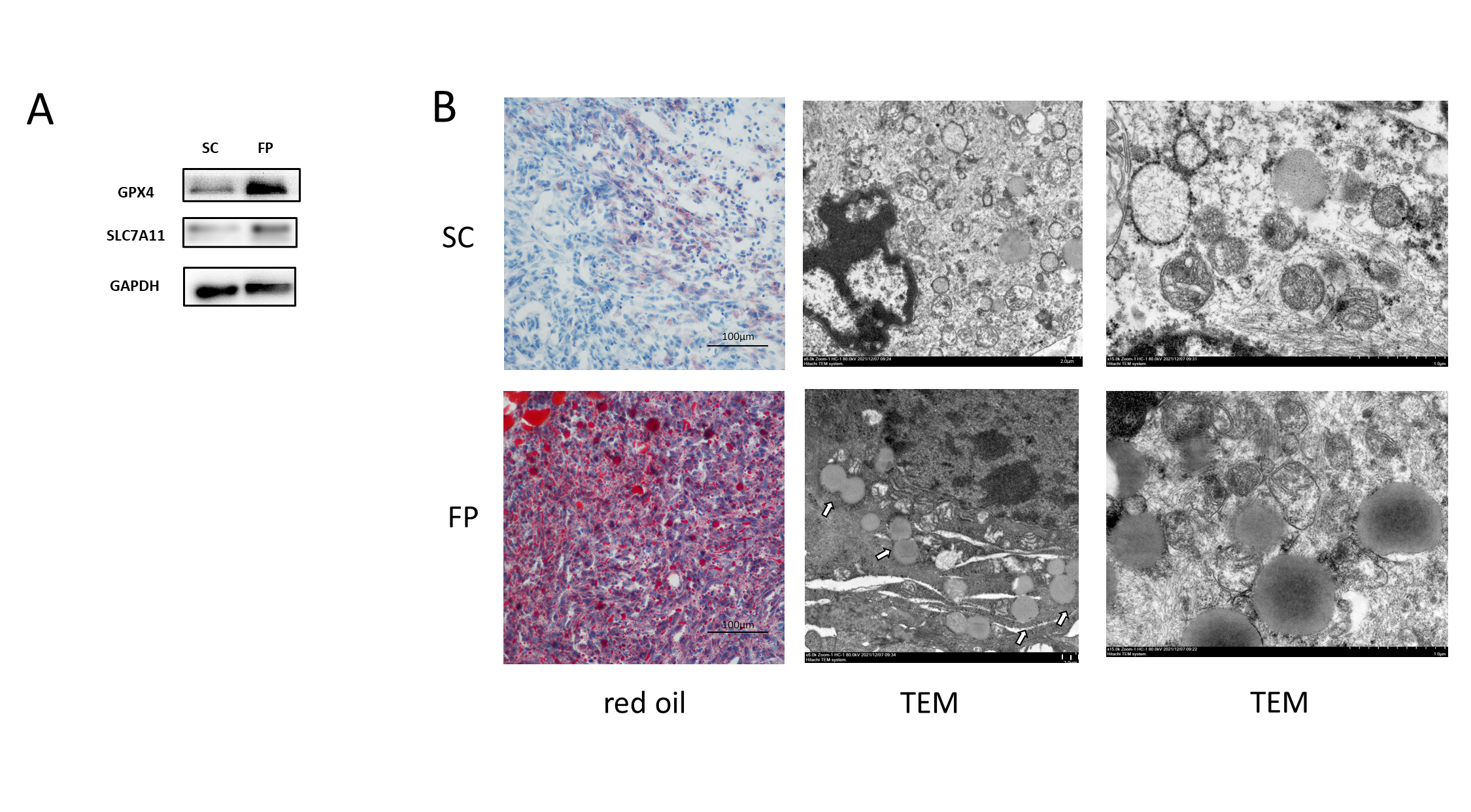

Supplement: Supplementary file 6 — Additional file 6 Fig. S6. Supporting figures of mice tumor tissue. A Protein levels of GPX4 and SLC7A11 were analyzed by western blotting in tumor of SC and FP group treated with SAS. B Adipose infiltration levels tested by Oil Red staining as well as TEM of resected tumor in FP and SC group treated with SAS. [file 13045_2022_1297_MOESM6_ESM.tif]
