# Supplementary material for: Mammary adipocytes protect triple-negative breast cancer cells from ferroptosis
Source: J Hematol Oncol. 2022 Jun 3;15:72. doi: 10.1186/s13045-022-01297-1 (PMC9164506; doi:10.1186/s13045-022-01297-1)
Supplement: Supplementary file 9 — Additional file 9 Material and Methods. [file 13045_2022_1297_MOESM9_ESM.docx]

Cell lines and reagents

BT549 and MDA-MB-231 cells were purchased from Chinese Academy of Sciences Shanghai Branch Cell Bank (Shanghai, China). BT-549 cells were maintained in RPMI-1640 medium (Gibco, USA) supplemented with 10% fetal bovine serum at 37°C in a humidified incubator containing 5% CO2. MDA-MB-231 cells were maintained in Leibovitz’s L15 medium (Gibco, USA) supplemented with 10% fetal bovine serum (Gibco, USA) at 37°C in a humidified incubator without CO2.

Adipose tissue samples were obtained with informed consent from patients at Fudan University Shanghai Cancer Center. The mature adipocytes were isolated and harvested as previous study[1, 2]. In short, fresh adipose tissues were crushed on ice and digested with type I collagenase (Sigma, Germany) under gentle agitation at 37°C for 60 min. Then we use FBS to terminate enzyme activity. The suspensions were centrifuged at 300g for 10 min and the upper layer was collected and transferred in culture flask. Next, we fulfilled the flask using DMEM/F12 (Life, USA) with 10% FBS, put it invertedly at 37°C in a humidified incubator containing 5% CO2. The flask was inverted again after adhesion of adipocytes.

All chemicals were purchased from Sigma-Aldrich unless stated otherwise.

Coculture, cell viability, and colony formation assays

For co-culture system, MDA-MB-231 and BT-549 cells (1*10^5^) were seeded in the bottom chamber and mature adipocytes (5*10^4^) were seeded in the top chamber of the co-culture system using 6-well Transwell plates (0.4μm pore size). In indicated experiments, cells were treated or not during co-culture system for 3 days and were collected for further experiments.

For cell viability tests, cells at appropriate density per well were plated in 96-well plates for 24 h adherent. Then cells were treated with chemicals (DMSO, SAS, ferrostatin-1, etc.) at indicated concentrations for 24-48 hours, followed by medium replacement of 100μL fresh medium containing 10 μL Cell Counting Kit-8 (CCK8) reagent (Beyotime, China) and were incubated for 1 h at 37°C. Cell viability was measured at 450 nm absorbance using a Universal Microplate Reader (BIO-TEK Instruments, USA) and calculated according to instructions.

For cell plate colony formation assay, cells with appropriate density per well were incubated in 6-well plates for 24 h. Then cells were treated with chemicals (DMSO, SAS, ferrostatin-1, etc.) at appropriate concentrations at 37 °C for 24-48 hours, followed by incubation without ferroptosis inducers for 1-2 weeks. Next, the dishes were washed, fixed with 4% paraformaldehyde (Sigma, USA) and stained with crystal violet (Beyotime, China). The morphology of cell colonies was recorded via photo imaging.

Lipid peroxidation assay

Cells with appropriate density per well were seeded in 6-well or 12-well plates for 24h, followed by treatment of indicated chemicals. Then cells were washed and incubated with 2μM BODIPY™ 581/591 C11 (Thermo Fisher Scientific, USA) for 30 min at 37°C. Subsequently, cells were washed and harvested by trypsinization (Gibco, USA) or direct observed by fluorescence microscopy. Then the harvested cells were resuspended in 500 μL of fresh PBS (Gibco, USA) by centrifugation. The fluorescence intensity of cells was measured using FL1 channel of flow cytometry (FC500 MPL, Beckman Coulter, USA) with a live cells filter. The relative lipid peroxidation level is measured through the cell percentage gated relied on the fluorescence intensity of FL1 channel. Data analysis was conducted using the FlowJo Software.

Transmission electron microscopy (TEM)

Cells were scraped and collected in 1.5mL Eppendorf (EP) tube (1*10^6) and tumor tissues were cut into 1mm3 size, followed by fixation using TEM Fixative (Servicebio, China) for at least 24h. Next, cells or tissues were post-fixed in 1% OsO4 in 0.1 M phosphate buffer (pH 7.4), dehydrated in gradual ethanol (30–100%), embedded in EMBed 812 (SPI, USA) and polymerized in 65°C for 48h. Then resin blocks were cut to 60-80nm thin on the ultra-microtome (Leica UC7, Leica, Germany), fished out onto the 150 meshes cuprum grids with formvar film and stained by 2% uranium acetate and 2.6% lead citrate. Subsequently, the cuprum grids were observed and took images under TEM (HT7800, HITACHI, Japan).

Oil Red staining

For Oil Red staining, cells in culture or tissue slices were fixed in fat fixative solution (Servicebio, China) for 15 minutes, stained with Oil Red solution (Servicebio, China) for 8-10 min in the dark, washed and immersed in 60% isopropanol for differentiation, washed and counterstained with hematoxylin (Servicebio, China). Then the slices were sealed and observed with microscope inspection, image acquisition and analysis.

Free fatty acid assay

The free fatty acid was detected by free fatty acid assay kit (Sigma, USA). Briefly, cell supernatants and palmitic acid standards were collected and put into a 96-well plate (50 μL). Then 2μL of ACS Reagent was added and incubated, followed by addition of 50μL of the Master Reaction Mix. After incubation, the absorbance at 570 nm (A570) was measured. The amount of fatty acids present in the samples was determined from the standard curve.

Western blot

Western blotting was performed according to a standard protocol of a previously described procedure[3]. The primary antibodies anti-ACSL3 (Abcam, 1:1000), anti-GPX4 (Proteintech, 1:1000), anti-SLC7A11 (Proteintech, 1:1000) and anti-GAPDH (Cell Signaling Technology, 1:1000) were used. The secondary antibody horseradish peroxidase-conjugated anti-rabbit IgG (Cell Signaling Technology, 1:5000) were used. Proteins were visualized using the ECL western blotting substrate (Thermo Fisher Scientific, USA).

Quantitative real-time PCR (qRT-PCR)

qRT-PCR was performed as previously described [36, 37]. Briefly, we used TRIzol reagent (Invitrogen, USA) to extract total RNA from cells. Then RNA was reversely transcribed into cDNA using cDNA Synthesis Kit (TaKaRa, Japan) and qRT-PCR was performed in use of SYBR Premix Ex TaqII (TaKaRa, Japan) on the RocheLightCycler 480 II with software (Roche, Switzerland). The 2^−ΔΔCt^ was used to represent relative expression of target genes using GAPDH as a reference gene. The forward primer of ACSL 3 was 5′- GCCGAGTGGATGATAGCTGC -3′, and the reverse primer was 5′- ATGGCTGGACCTCCTAGAGTG -3′.

Construction of ACSL3-knockdown cell lines

The shRNAs targeting the ACSL3 in PHY-304 lentiviral vectors and a negative control were constructed by Hanyin Co. (Shanghai, China). Target Sequences of shRNA targeting ACSL3 were: 5′- GGCCCATGTTCTAGAATTA -3′ (shACSL3-1), 5′- GGAAGGTGGATACTTTAAT -3′ (shACSL3-2). To obtain stable cell lines, MDA-MB-231 and BT-549 cells were seeded in six‑well plates and infected with shRNA virus and polybrene the following day according to the manufacturer’s instruction. Cells that stably express lentiviral shRNAs were selected with 1 µg/ml puromycin for 7-14 days to establish stable cell lines: 231-shNC, 231-shACSL3-1, 231-shACSL3-2, 549-shNC, 549-shACSL3-1, 549-shACSL3-2. The efficiency of ACSL3 knockdown and overexpression was confirmed by qRT‑PCR.

Animal experiments

Animal experiments were conducted in accordance with a protocol reviewed and approved by the Animal Care Committee of Fudan University Shanghai Cancer Center (Approval No. FUSCC-IACUC-S20220001, Shanghai, China). Female 4–6-week-old BALB/c nude mice were bought from Shanghai Model Organisms (Shanghai, China) and housed in a sterile animal room. 1*10^6^ MDA-MB-231 cells were injected into fat pad (FP) of left groin and right back subcutaneously (SC) of mice to grow tumors up to approximately 100 mm^3^. Mice were then intragastrically delivered SAS (400 mg/kg) until the endpoint indicated in the corresponding figures. Mice were monitored and measured daily.

Statistical analysis

The study results are presented as mean ± standard deviation (SD) for three independent experiments. Student’s t-test was used for comparison between groups. OS curves were conducted using Kaplan–Meier analyses and compared using log-rank test. All statistical analyses were performed using the GraphPad Prism® 8.0 software or R software (R i386 4.0.2). P < 0.05 (two-side) was considered statistically significant.

**References:**

[1]. Harms, M.J., et al., Mature Human White Adipocytes Cultured under Membranes Maintain Identity, Function, and Can Transdifferentiate into Brown-like Adipocytes. Cell Reports, 2019. 27(1): p. 213-225.e5.

[2]. Wang, Z., et al., Effect of advanced glycosylation end products on apoptosis in human adipose tissue-derived stem cells in vitro. Cell Biosci, 2015. 5: p. 3.

[3]. Chen, S., et al., Regulation of microtubule stability and organization by mammalian Par3 in specifying neuronal polarity. Dev Cell, 2013. 24(1): p. 26-40.
